# The concurrent validity of the Lund University Checklist for Incipient Exhaustion and the Karolinska Exhaustion Disorder Scale: a replication study

**DOI:** 10.1186/s13104-023-06589-4

**Published:** 2023-11-09

**Authors:** Roger Persson, Kai Österberg, Jonas Björk

**Affiliations:** 1 Department of Psychology, Lund University, Lund, SE-22100 Sweden; 2 Department of Laboratory Medicine, Division of Occupational and Environmental Medicine, Lund University, Lund, SE-22100 Sweden; 3 Clinical Studies Sweden, Forum South, Skåne University Hospital, Lund, SE-22100 Sweden

**Keywords:** Burnout, Exhaustion disorder, KEDS, LUCIE, Mental health, Screening, Stress, Work

## Abstract

**Objective:**

As part of our research on Swedish school principals, we examined the concurrent validity between the Karolinska Exhaustion Disorder Scale (KEDS) and the Lund University Checklist for Incipient Exhaustion (LUCIE) in a cross-sectional study sample (N = 2670). Specifically, we examined: (a) to what extent LUCIE and KEDS identified the same individuals and their level of agreement, and (b) to what extent the present observations among school-principals agreed with previous observations made in a highly educated and healthy study sample drawn from the general population.

**Results:**

Depending on established cut-points on LUCIE, the Kappa agreement (*K*) between LUCIE and KEDS varied between fair (*K* = 0.34 [95% Confidence Interval = 0.30–0.38]) and moderate (*K* = 0.54 [95% Confidence Interval = 0.51–0.58]). While the instruments did not always identify the same individuals, the most reasonable comparison between KEDS and LUCIE was achieved when the cut-off on LUCIE was made between step two and step three. The results essentially replicated our previous results observed in a highly educated and healthy study sample drawn from the general population. The level of agreement suggests that KEDS and LUCIE scores are supplementary rather than interchangeable. Thus, individual result from KEDS and LUCIE are probably best understood in dialogue with the person screened.

**Supplementary Information:**

The online version contains supplementary material available at 10.1186/s13104-023-06589-4.

## Introduction

Constructing instruments and making measurements are fundamental activities in empirical sciences. And as verified by the large number of screening instruments that are available for the screening of anxiety and depression symptoms, screening instruments are often developed for identical, or highly similar, purposes, and many times assumed to be interchangeable [1]. However, to interpret tests scores from screening instruments that assess the same, or highly similar, phenomena, it is essential to know how they relate to one another in various groups and/or contexts. A too large correspondence seem to propose that either of the instruments is superflous. Oppositely, a too low correspondence seem to propose that the instruments assess unrelated phenomena, or that one, or both, instruments lack sufficient precision.

In 2005, to contribute to the mitigation of work-related mental health problems (e.g., anxiety, burnout, depression and exhaustion disorder) that have troubled many governments during the last decades [2–4], the Swedish National Board of Health and Welfare (NBHW) implemented the diagnosis Exhaustion Disorder (ED: F43.8 A) in the tenth revision of the International Classification of Diseases (ICD-10-SE) [5–7]. In brief, to attain the ED diagnosis, the patient needs to experience significant suffering, and/or a reduced functioning at work or social situations, while reporting a symptomatology of bodily and mental complaints during a minimum of two weeks, related to excessive fatigue in response to one or more identifiable stressors of a duration of at least six months. The patient must also be devoid of other medical conditions that might explain fatigue (e.g., depression, diabetes, general anxiety, heart disease, thyroid disease, substance abuse etc.). Observably, the similarities between ED and the concepts of burnout [8], or “clinical burnout” [9], are obvious, and screening questionnaires for ED and burnout tend to exhibit a strong positive association [10, 11].

Following the implementation of the ED diagnosis, the Karolinska Exhaustion Disorder Scale (KEDS) [12] and the Lund University Checklist for Incipient Exhaustion (LUCIE) [10, 13, 14] were developed to assist in the detection, monitoring and/or treatment of ED. And it is not uncommon that these screening inventories are used concurrently in research or clinical practice [10, 15–17]. However, differences in their (a) emphasis (manifest signs [KEDS] versus incipient signs [LUCIE]), (b) response formats (a seven step Likert scale with alternating verbal anchors [KEDS] versus a four step Likert scale [LUCIE]), (c) time frames (two weeks [KEDS] versus four weeks [LUCIE]), and (d) scoring procedures, suggest that KEDS and LUCIE might be assumed to give results that are supplementary rather than interchangeable.

We have previously, in a study sample drawn from the general population, benchmarked KEDS and LUCIE with each other and other inventories relating to stress and burnout, [10, 11, 13, 14], showing that KEDS and LUCIE are systematically positively related and that the Kappa agreement is fair to moderate [10]. In the present study, we sought to replicate our previous observations by comparing KEDS and LUCIE in a new study sample entailing Swedish school principals. From previous analyses, we knew that school principals had circa a twice as high prevalence proportion of stress and exhaustion symptoms [17] than our previous population sample [10]. In addition, the school principals had clearly higher prevalence proportions than what has been observed among leaders and employees in a politically governed regional organisation [18] and among occupational therapists [19]. Although comparisons of prevalence proportions across study samples should be made with caution due to varying selection procedures, the ostensibly higher proportion of school principals with exhaustion signs generate a beneficial condition for examining the agreement between KEDS and LUCIE. The research questions were:

- To what extent do LUCIE and KEDS identify the same individuals and what is the level of agreement between the instruments?
- To what extent do the present observations among school principals agree with our previous observations of Kappa agreement in a highly educated and healthy population sample?

## Methods

### Participants and study design

The present study targeted Swedish principals at all school levels. In the absence of an accessible official register of occupationally active school principals, we recruited participants via an e-mail list that had a nationwide reach and covered principals who during the period 2008–2017 had participated in training programmes funded and arranged by the Swedish National Agency for Education and run by different universities in Sweden. In the present study, the first response from principals and assistant principals to an online survey issued in 2018 (n = 2274) and 2019 (n = 1959) [20] were used (n = 2670). Of these, 2219 responded in 2018 and 451 in 2019. The participants also had to work at least 50% of full-time in compulsory schools (46%), pre-schools (27%), upper secondary schools (15%), adult education (7%), or pre- and compulsory schools (5%). Their mean age was 49.4 years (SD = 7.3 years). Most participants were women (78%) and employed by a municipality (77%).

### Measures

The LUCIE and KEDS inventories have been published [10, 12].

*The Lund University Checklist for Incipient Exhaustion* (LUCIE) entails 28 items covering six domains that build two supplementary scales: the *Stress Warning Scale (SWS)* and *Exhaustion Warning Scale (EWS)* [10]. Both scales ranges from 0 to 100 points. Cronbach’s alpha for the 28 LUCIE items was 0.94. And the median spearman rank order correlation between the SWS score and the individual LUCIE items was rho = 0.58 (Min = 0.44; Max = 0.70). For the EWS score, the median correlation with individual LUCIE items was rho = 0.47 (Min = 0.33; Max = 0.58). Following the manual, the SWS and EWS scores were categorized and combined. Specifically, SWS entails the three levels: Green (≤ 17.00), Yellow (17.01 to 38.50), and Red ≥ 38.51) whereas EWS entails two levels: Green (≤ 21.49) and Red (≥ 21.50). Using the pre-defined cut-off scores on each scale, the SWS and EWS are in practice combined into a four-step ladder of incremental stress symptomatology in which the combinations (a) SWS Green and EWS Red, and (b) SWS Yellow and EWS Red, are not possible to obtain or very rare, respectively. The four steps are:

Step 1-GG (SWS green zone and EWS green zone), indicating no signs of stress;

Step 2-YG (SWS yellow zone and EWS green zone), indicating possible, or weak signs, of stress;

Step 3-RG (SWS red zone and EWS green zone), indicating mild to moderate lasting stress symptoms, but less severe than ED;

Step 4-RR (SWS red zone and EWS red zone), indicating severe stress signs and possible ED.

*The Karolinska Exhaustion Disorder Scale* (KEDS) comprises nine items that refer to the past two weeks, covering the following nine domains: (a) ability to concentrate, (b) memory, (c) physical stamina, (d) mental stamina, (e) recovery, (f) sleep, (g) hypersensitivity to sensory impressions, (h) experience of demands, and (i) irritation and anger [12]. Each item is responded to on a 7-point scale (0–6). Descriptive verbal phrases, tailored for each separate item on a scale of increasing severity describe the corresponding domain-specific symptomatology, serving as anchors for the scale steps 0, 2, 4 and 6. Higher values reflect more severe symptoms, and the sum score (0 to 54) is used as an outcome, with a score ≥ 19 indicating possible ED [12, 21]. Cronbach’s alpha was 0.88. And the median spearman rank order correlation between the continuous total KEDS sum score and individual KEDS items was rho = 0.70 (Min = 0.67; Max = 0.80).

### Data management and statistical analysis

Using the IBM SPSS software version 29, we applied non-parametric and parametric testing (two-tailed alpha level ≤ 0.05). Spearman rank correlations and Kappa statistics (both with bootstrap estimated 95% confidence intervals [95% CI] to compensate for ties) were used to estimate the degree of association and agreement between LUCIE and KEDS. For purpose of analysis, and to create symmetry between LUCIE and KEDS categories, we examined the changes in the Kappa statistics following the use of various cut-off scores for LUCIE. Sensitivity analyses were conducted by excluding individuals, by analysing data separately for men and women, and for subgroups defined by the year of the first response (i.e., 2018 or 2019). Landis and Koch criteria for judgment of the Kappa statistics were used: *K* 0–20 = poor; *K* ≤ 21–40 = fair; *K* ≤ 41–60 = moderate; *K* ≤ 61–80 = substantial; *K* > 80 = Good [22].

## Results

Table 1 shows the proportion of individuals within a specific LUCIE category that reports above or below the exhaustion indication level in KEDS (Table 1). Descriptive data, and measures of the associations between the continuous LUCIE SWS and EWS scale scores and the KEDS sum scores, are presented in Additional file 1 (Table S1 and S2 and Figures S1 to S3).

**Table 1:** Descriptive cross-tabulation. The table shows the proportion of subjects within each Lund University Checklist of Incipient Exhaustion (LUCIE) category (Column %) that had either a normal score, or an exhaustion indication, on the Karolinska Exhaustion Disorder Scale (KEDS).

	LUCIE									
	Step 1-GG		Step 2-YG		Step 3-RG		^1^Step 4-RR		Total	
**KEDS**	%	N	%	N	%	N	%	N	%	N
^2^Normal	94.7	1242	66.4	451	36.7	147	15.1	42	70.5	1882
^3,4^Exhaustion	5.3	70	33.6	228	63.3	254	84.9	236	29.5	788
**Total**	100	1312	100	679	100	401	100	278	100	2670

Note: ^1^The rare combination of SWS yellow + UWS red was included in LUCIE Step 4-RR (n = 14)

^2^The row percentages for KEDS normal are 66% (Step 1-GG), 24% (Step 2-YG), 7.8% (Step 3-RG), and 2.2% (Step 4-RR)

^3^The row percentages for KEDS exhaustion are 8.9% (Step 1-GG), 28.9% (Step 2-YG), 32.2% (Step 3-RG), and 29.9% (Step 4-RR)

^4^KEDS score ≥ 19.0

Table 2 shows the degree of Kappa agreement between LUCIE and KEDS. The lowest agreement was observed when making a cut-off between LUCIE step 3-RG and step 4-RR (*K* = 0.34). The highest agreement was observed when making a cut-off between LUCIE step 2-YG and step 3-RG (*K* = 0.54). At this cut-off point, the prevalence proportions for LUCIE and KEDS were 25.4% and 29.5%, respectively. Subsequent sensitivity analyses, in which individuals on step 2-YG and 3-RG were excluded, showed as expected a higher degree of agreement (i.e., *K*´s between 0.70 and 0.77) and revealed similar patterns of Kappa agreement for men and women and for subgroups of participants defined by their year of first response (see Additional file 2, Table S3 and S4).

**Table 2:** Estimated Kappa agreement between the Lund University Checklist of Incipient Exhaustion (LUCIE) and the Karolinska Exhaustion Disorder Scale (KEDS) at different LUCIE cutoff points and by excluding identifications in the middle range of LUCIE (sensitivity analysis)

^2^LUCIE		KEDS						Kappa (95% ^1^CI) [^3^Reference data]
		Normal		Exhaustion		Total		
		N	%	N	%	N	%	
**Gliding cut-off point**								
Step 1 GG **Vs.** Step 2-YG, 3-RG, 4-RR	No	1242	66.0	70	8.9	1312	49.1	0.47 (0.44–0.50)
	Yes	640	34.0	718	91.1	1358	50.9	[0.36 (0.32–0.42)]
	Total	1882	100.0	788	100.0	2670	100.0	[N = 1339]
Step 1-GG, 2-YG **Vs.** Step 3-RG, 4-RR	No	1693	90.0	298	37.8	1991	74.6	0.54 (0.51–0.58)
	Yes	189	10.0	490	62.2	679	25.4	[0.48 (0.41–0.55)]
	Total	1882	100.0	788	100.0	2670	100.0	[N = 1339]
Step 1-GG, 2-YG, 3-RG **Vs.** Step 4-RR	No	1840	97.8	552	70.1	2392	89.6	0.34 (0.30–0.38)
	Yes	42	2.2	236	29.9	278	10.4	[0.28 (0.20–0.36)]
	Total	1882	100.0	788	100.0	2670	100.0	[N = 1339]
**Excluding steps**								
Step 1-GG **Vs.** Step 3-RG, 4-RR	No	1242	86.8	70	12.5	1312	65.9	0.70 (0.66–0.73)
	Yes	189	13.2	490	87.5	679	34.1	[0.60 (0.52–0.66)]
	Total	1431	100.0	560	100.0	1991	100.0	[N = 1062]
LUCIE Step 1-GG **Vs.** Step 4-RR	No	1242	96.7	70	22.9	1312	82.5	0.77(0.72–0.81)
	Yes	42	3.3	236	77.1	278	17.5	[0.60 (0.49–0.70)]
	Total	1284	100.0	306	100.0	1590	100.0	[N = 945]

Note: ^1^ CI = Confidence Interval (calculated with bootstrap estimation)

^2^The rare combination of SWS yellow + UWS red was included in LUCIE Step 4-RR  (n = 14)

^3^Data derived from Table 4 in Persson et al. (2016) that used a highly educated healthy population sample drawn from the general population. Data was originally published in BMC Public Health [Persson et al., 2016]

Table 3 presents a comparison of the continuous mean and median KEDS sum scores across the four LUCIE steps of incremental signs of exhaustion. Descriptive sensitivity analyses for subgroups of men and women indicated that the pattern of mean and median KEDS sum scores were similar (see Additional file 3, Table S5).

**Table 3:** Descriptive Karolinska Exhaustion Disorder Scale (KEDS) scores (M), standard deviations (SD), median scores (Mdn), and accompanying 95% Confidence intervals (CI) across the four Lund University Checklist of Incipient Exhaustion (LUCIE) categories

^2^LUCIE	Total sample (N = 2670)								
		Age (years)		^1^KEDS score					
	N	M	SD	M	SD	95% CI		Mdn	95% CI
Step 1-GG	1312	49.4	7.4	9.1	5.4	8.8–9.4		9.0	9.0–10.0
Step 2-YG	679	49.7	7.1	15.9	5.8	15.4–16.3		16.0	16.0–17.0
Step 3-RG	401	48.9	7.6	21.0	6.2	20.4–21.6		20.0	20.0–21.0
^2^Step 4-RR	278	49.2	7.3	26.9	8.0	26.0-27.9		27.0	26.0–28.0

Note: ^1^ A KEDS score ≥ 19 indicates plausible exhaustion disorder. The score can vary between 0 to 54 points

^2^The rare combination of SWS yellow + UWS red was included in LUCIE Step 4-RR (n = 14)

^3^ Six principals did not disclose their gender

## Discussion

In essence, the pattern of Kappa estimates between LUCIE and KEDS in the present sample of Swedish principals and assistant principals can be said to replicate our previous observations in a study sample entailing highly educated and healthy individuals drawn from the general population [10]. However, and even if the Kappa estimates were consistently higher in the present study sample (see Table 2), the Kappa estimates had overlapping confidence intervals. Overall, the pattern of results from our present study and previous studies suggests that the concurrent validity of LUCIE and KEDS is a stable phenomenon across study samples, contexts, and gender.

That the Kappa agreement between LUCIE and KEDS at best amounts to “moderate” according to the Landis and Koch criteria [22] is not very surprising in view of the aforementioned differences in emphasis, item formats, covered time frames and that LUCIE makes a more fine-grained classification of early stress and exhaustion signs across four categories whereas KEDS uses two categories (i.e., indication/no-indication). Thus, to estimate the Kappa agreement, we dichotomized the LUCIE categories by applying various cut-off points. In so doing, we observed that the best agreement was achieved using a cut off between LUCIE step 2-YG and step 3-RG. Furthermore, as shown when computing means and median KEDS sum scores for each of the LUCIE categories, there is a clear escalating pattern. And that both the mean and median KEDS score falls just above the cut-off score for an exhaustion indication (≥ 19.0) at LUCIE step 3-RG, suggest that the most reasonable comparison between KEDS and LUCIE is achieved when merging step 3-RG and Step 4-RR in LUCIE. This observation is also in alignment with the general recommendation to use step 3-RG as the first significant indicator of stress symptoms of a clinical magnitude [15].

Noticeably, if excluding step 2-YG and/or step 3-RG the agreement between LUCIE and becomes substantial according to Landis and Kochs criteria [22]. Thus, apart from the reduction in categories it is also clear that the higher prevalence proportion of stress and exhaustion symptoms in the present study sample will influence the Kappa estimates. The higher prevalence proportion of exhaustion symptoms in the present study sample [17, 20] when compared to our previous population sample [10] is also a likely contributor to the consistently higher Kappa agreement in the present sample even if the 95% CI consistently overlap.

## Conclusions

The present results essentially replicate our previous observations among highly educated and healthy individuals drawn from the general population. The fair to moderate Kappa agreement between KEDS and LUCIE suggests that these instruments might be supplementary rather than interchangeable. Thus, while the participants’ concurrent ratings in LUCIE and KEDS are systematically, logically, and consistently associated with each other, the instruments do not necessarily identify the same individuals as being stressed and/or exhausted. This underlines that on the individual level, the results from these screening instruments are best regarded as indicators of possible illness that needs to be understood in a broader context and in a confident dialogue with the person screened.

## Limitations

All participants had a manager position, long education, and were in active full-time work to an extent of at least 50% of full-time. In the absence of a gold standard for assessing exhaustion disorder symptoms, it should be noted that our analyses only confirm the concurrent agreement between the two instruments. And because KEDS favours a dichotomized approach to identify possible exhaustion cases, comparisons with LUCIE, that favours a partitioning into four classes, become more difficult and less intuitively straightforward. In addition, the prevalence proportions of stress and exhaustion warnings in both LUCIE and KEDS were higher in this occupational sample than has been previously observed in the general population and in other occupational samples. Generalizations to other occupations and/or segments of the population (e.g., people with diagnosed ED) should be made with caution.

### Electronic supplementary material

Below is the link to the electronic supplementary material.

Supplementary Material 1

Supplementary Material 2

Supplementary Material 3

## Data Availability

Consistent with the study protocol approved by the Regional Ethical Review Board, anonymized data is stored locally at the Division of Occupational and Environmental Medicine, Lund University, Lund, Sweden. Because the participants (in accordance with the approved study protocol) were guaranteed that the crude data should not be published on the internet, access to data will only be granted to eligible researchers wanting to audit our research.

## Abbreviations

CI: Confidence interval
KEDS: Karolinska Exhaustion Disorder Scale
LUCIE: Lund University Checklist for Incipient Exhaustion
NBHW: Swedish National Board of Health and Welfare
ED: Exhaustion Disorder
EWS: Exhaustion Warning Scale
Step 1-GG: SWS Green zone and EWS Green zone
Step 2-YG: SWS Yellow zone and EWS Green zone
Step 3-RG: SWS Red zone and EWS Green zone
Step 4-RR: SWS Red zone and EWS Red zone
SWS: Stress Warning Scale
