# Supplementary material for: The concurrent validity of the Lund University Checklist for Incipient Exhaustion and the Karolinska Exhaustion Disorder Scale: a replication study
Source: BMC Res Notes. 2023 Nov 9;16:325. doi: 10.1186/s13104-023-06589-4 (PMC10636803; doi:10.1186/s13104-023-06589-4)
Supplement: Supplementary file 1 — Supplementary Material 1 [file 13104_2023_6589_MOESM1_ESM.docx]

**ADDITIONAL FILE 1**

In this file we present descriptive data (Table S1), Spearman rank order correlations (Table S2), and bivariate scatterplots (Figure S1 to S3), for the Stress Warning Scale (SWS) (0-100), the Exhaustion Warning Scale (EWS), and the Karolinska Exhaustion Disorder Scale (KEDS).

**Table S1.** Descriptive Means (M), standard deviations (SD), median scores (Mdn), and the 25^th^ and 75^th^ percentile scores for the Stress Warning Scale (SWS) and the Exhaustion Warning Scale (EWS) in the Lund University Checklist of Incipient Exhaustion (LUCIE) and the Karolinska Exhaustion Disorder Scale (KEDS).

| **Scale** | **N** |  | **M** | **SD** |  | **Mdn** | **25^th^** | **75^th^** |  |
| --- | --- | --- | --- | --- | --- | --- | --- | --- | --- |
| SWS (0-100) | 2670 |  | 23.8 | 22.5 |  | 18.2 | 4.2 | 38.3 |  |
| EWS (0-100) | 2670 |  | 6.7 | 12.2 |  | 0.0 | 0.0 | 8.9 |  |
| KEDS (0-54) | 2670 |  | 14.5 | 8.5 |  | 14.0 | 8.0 | 20.0 |  |

Note: The SWS and EWS can take on scores between 0 and 100. The KEDS sum score can take on scores between 0 and 54.

**Table S2.** Spearman rank order correlations for the Stress Warning Scale (SWS) and the Exhaustion Warning Scale (EWS) (0-100) in the Lund University Checklist of Incipient Exhaustion (LUCIE) and the Karolinska Exhaustion Disorder Scale (KEDS).

| **Scale** | **N** | **SWS** | **EWS** | **KEDS** |
| --- | --- | --- | --- | --- |
| SWS (0-100) | 2670 | -- | 0.73** (0.71-0.74) | 0.75**(0.73-0.77) |
| EWS (0-100) | 2670 |  | -- | 0.54** (0.51-0.57) |
| KEDS (0-54) | 2670 |  |  | -- |

Note: ** = p-value < 0.001. The SWS and EWS can take on scores between 0 and 100. The KEDS sum score can take on scores between 0 and 54.

**Figure S1**

**
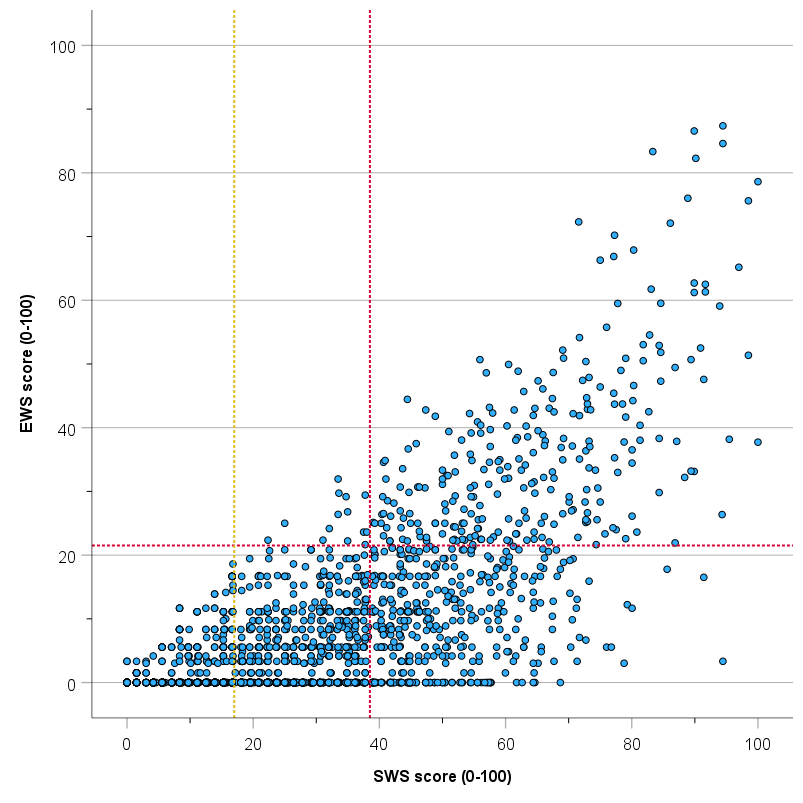
**

**Figure S1.** Descriptive bivariate scatterplot between the LUCIE Stress Warning Scale (SWS) and the LUCIE Exhaustion Warning Scale (EWS) (N=2670). The vertical yellow line indicates the cut off level between SWS Green and SWS Yellow (17.0 points). The vertical red line indicates the cut off level between SWS Yellow and SWS Red (38.5 points). The horizontal redline indicates the cut off level for EWS green and EWS Red (21.5 points).

**Figure S2**


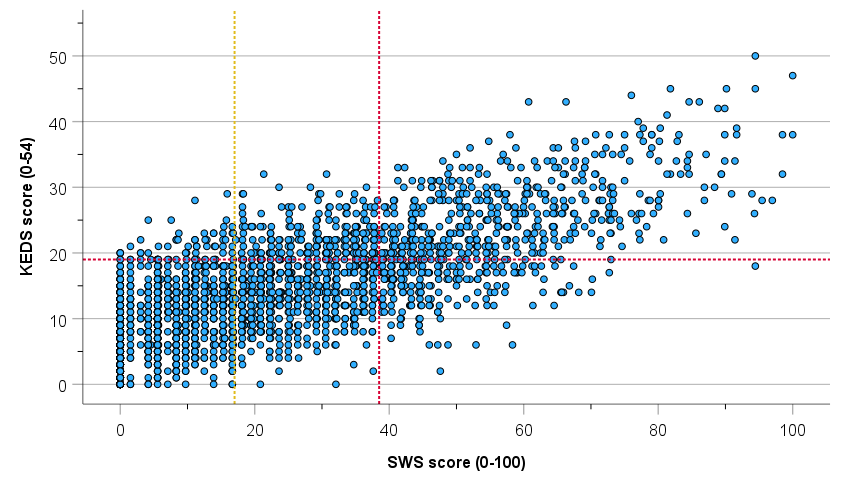


**Figure S2.** Descriptive bivariate scatterplot between LUCIE SWS scores and KEDS scores (N=2670). The vertical yellow line indicates the cut off level between SWS Green and SWS Yellow (17.0 points). The vertical red line indicates the cut off level between SWS Yellow and SWS Red (38.5 points). The horizontal redline indicates the cut off level for signs of exhaustion in KEDS (19.0 points).

**Figure S3**

**
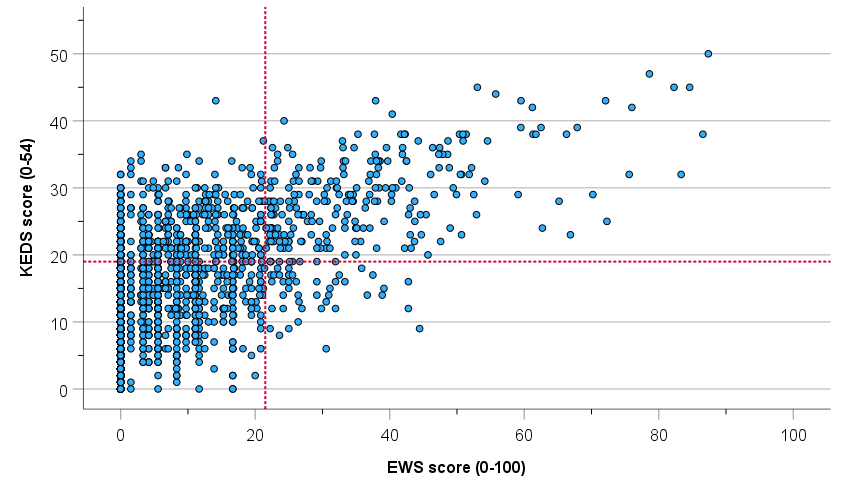
**

**Figure S3.** Descriptive bivariate scatterplot between LUCIE Exhaustion Warning Scale (EWS) scores and KEDS scores (N=2670). The vertical red line indicates the cut off level between EWS Green and EWS Red (21.5 points). The horizontal redline indicates the cut off level for signs of exhaustion in KEDS (19.0 points).
