# Supplementary material for: The concurrent validity of the Lund University Checklist for Incipient Exhaustion and the Karolinska Exhaustion Disorder Scale: a replication study
Source: BMC Res Notes. 2023 Nov 9;16:325. doi: 10.1186/s13104-023-06589-4 (PMC10636803; doi:10.1186/s13104-023-06589-4)
Supplement: Supplementary file 2 — Supplementary Material 2 [file 13104_2023_6589_MOESM2_ESM.docx]

**ADDITIONAL FILE 2**

In this file we present the results from two sensitivity analyses. The first analysis examines the Kappa agreement in subgroups of men and women (Table S3). The second analysis examines the Kappa agreement in subgroups arbitrarily defined by the participants year of entry in the study (Table S4).

**Table S3.** Estimated Kappa agreement for women and men with associated 95% confidence intervals (CI) between the Lund University Checklist of Incipient Exhaustion (LUCIE) and the Karolinska Exhaustion Disorder Scale (KEDS) at different LUCIE cut off points and by excluding identifications in the middle range of LUCIE.

|  | **Women** | | | | |  | | **Men** | | | | | |  |
| --- | --- | --- | --- | --- | --- | --- | --- | --- | --- | --- | --- | --- | --- | --- |
| ^1^**LUCIE** | **KEDS**  **(2-levels)** | | | | |  | | **KEDS**  **(2-levels)** | | | | | |  |
| **Gliding cut off point** | **N** | **Kappa** | | **95% CI** | |  | | **N** | | **Kappa** | | **95% CI** | |  |
| 1 – (**2, 3, 4)** | 2082 | 0.47 | | 0.43-0.51 | |  | | 582 | | 0.47 | | 0.41-0.54 | |  |
| 1, 2 – (**3, 4)** | 2082 | 0.53 | | 0.49-0.57 | |  | | 582 | | 0.59 | | 0.51-0.67 | |  |
| 1, 2, 3 – (**4)** | 2082 | 0.35 | | 0.31-0.39 | |  | | 582 | | 0.29 | | 0.19-0.37 | |  |
| **Excluding steps** |  |  |  | |  | |  | |  | |  | |  | |
| 1**, (~~2~~),** 3, 4 | 1535 | 0.69 | | 0.65-0.73 | |  | | 451 | | 0.72 | | 0.63-0.79 | |  |
| 1, **(~~2, 3~~),** 4 | 1227 | 0.76 | | 0.72-0.81 | |  | | 358 | | 0.76 | | 0.64-0.86 | |  |

Note: Confidence intervals calculated with bootstrap estimation.

^1^The rare combination of SWS yellow + UWS red was included in LUCIE Step 4-RR (N=14).

**Table S4.** Estimated Kappa agreement with associated 95% confidence intervals (CI) for subgroups defined by year of entry (i.e., first participant data collected 2018 or 2019) between the Lund University Checklist of Incipient Exhaustion (LUCIE) and the Karolinska Exhaustion Disorder Scale (KEDS) at different LUCIE cut off points and by excluding identifications in the middle range of LUCIE.

|  | | **2018** | | | | |  | | **2019** | | | | | | | |  |
| --- | --- | --- | --- | --- | --- | --- | --- | --- | --- | --- | --- | --- | --- | --- | --- | --- | --- |
| ^1^**LUCIE** | | **KEDS**  **(2-levels)** | | | | |  | | **KEDS**  **(2-levels)** | | | | | | | |  |
| **Gliding cut off point** | **N** | | **Kappa** | | **95% CI** | |  | | | **N** | | **Kappa** | | **95% CI** | |  |  |
| 1 – (**2, 3, 4)** | 2219 | | 0.46 | | 0.43-0.50 | |  | | | 451 | | 0.52 | | 0.43-0.59 | |  |  |
| 1, 2 – (**3, 4)** | 2219 | | 0.54 | | 0.50-0.58 | |  | | | 451 | | 0.56 | | 0.47-0.64 | |  |  |
| 1, 2, 3 – (**4)** | 2219 | | 0.34 | | 0.30-0.38 | |  | | | 451 | | 0.35 | | 0.26-0.44 | |  |  |
| **Excluding steps** |  | |  |  | |  | |  | | |  | |  | |  | | |
| 1**, (~~2~~),** 3, 4 | 1650 | | 0.69 | | 0.66-0.73 | |  | | | 341 | | 0.73 | | 0.65-0.80 | |  |  |
| 1, **(~~2, 3~~),** 4 | 1309 | | 0.77 | | 0.72-0.81 | |  | | | 281 | | 0.76 | | 0.65-0.85 | |  |  |

Note: Confidence intervals calculated with bootstrap estimation.

^1^The rare combination of SWS yellow + UWS red was included in LUCIE Step 4-RR (n=14).
