# Supplementary material for: The concurrent validity of the Lund University Checklist for Incipient Exhaustion and the Karolinska Exhaustion Disorder Scale: a replication study
Source: BMC Res Notes. 2023 Nov 9;16:325. doi: 10.1186/s13104-023-06589-4 (PMC10636803; doi:10.1186/s13104-023-06589-4)
Supplement: Supplementary file 3 — Supplementary Material 3 [file 13104_2023_6589_MOESM3_ESM.docx]

**ADDITIONAL FILE 3**

In this file we present a descriptive account for how men (n=582) and women (n=2082) score on the Karolinska Exhaustion Disorder Scale (KEDS) and how the scores vary across the four category steps in the Lund University Checklist of Incipient Exhaustion (LUCIE).

**Table S5.** Descriptive Karolinska Exhaustion Disorder Scale (KEDS) mean sum scores (M), standard deviations (SD), median scores (Mdn), and accompanying 95% confidence intervals (CI) across the four Lund University Checklist of Incipient Exhaustion (LUCIE) categories for men and women.

| ^3^**Women** | | | | | | | | | | | | | |
| --- | --- | --- | --- | --- | --- | --- | --- | --- | --- | --- | --- | --- | --- |
|  |  | **Age (years)** | |  | **^1^KEDS score** | | | | | |  | | |
| ^2^**LUCIE** | **N** | **M** | **SD** |  | **M** | **SD** | **95% CI** |  | **Mdn** | **95% CI** | |  | |
| Step 1-GG | 990 | 49.6 | 7.2 |  | 9.3 | 5.6 | 8.9-9.6 |  | 9.0 | 9.0-10.0 | |  | |
| Step 2-YG | 547 | 49.8 | 7.0 |  | 16.2 | 5.8 | 15.7-16.7 |  | 16.0 | 16.0-17.0 | |  | |
| Step 3-RG | 308 | 49.6 | 7.4 |  | 21.0 | 6.4 | 20.3-21.7 |  | 20.0 | 20.0-21.0 | |  | |
| ^2^Step 4-RR | 237 | 49.2 | 7.3 |  | 27.1 | 8.1 | 26.1-28.1 |  | 28.0 | 28.0-29.0 | |  | |
| ^3^**Men** | | | | | | | | | | | | | |
|  |  | **Age (years)** | |  | **^1^KEDS score** | | | | | |  | | |
| ^2^**LUCIE** | **N** | **M** | **SD** |  | **M** | **SD** | **95% CI** |  | **Mdn** | **95% CI** | | |  |
| Step 1-GG | 318 | 48.6 | 7.9 |  | 8.6 | 5.0 | 8.0-9.1 |  | 8.0 | 8.0-9.0 | | |  |
| Step 2-YG | 131 | 49.4 | 7.5 |  | 14.4 | 5.7 | 13.4-15.4 |  | 14.0 | 13.0-16.0 | | |  |
| Step 3-RG | 93 | 46.6 | 7.8 |  | 20.9 | 5.4 | 19.8-22.0 |  | 20.0 | 19.0-22.0 | | |  |
| ^2^Step 4-RR | 40 | 48.8 | 7.8 |  | 25.9 | 8.1 | 23.3-28.5 |  | 24.5 | 22.0-31.0 | | |  |

^1^ A KEDS score ≥ 19 indicates plausible exhaustion disorder. The score can take on values from 0 to 54.

^2^The rare combination of SWS yellow + UWS red was included in LUCIE Step 4-RR (n=14).

^3^ Six school principals did not disclose their gender.
